# Does COVID-19 shock endanger the flows of FDI in OECD? Empirical evidence based on AMG panel estimator

**DOI:** 10.1186/s43093-022-00132-w

**Published:** 2022-07-07

**Authors:** Jamiu Olamilekan Badmus, Sodiq Olaide Bisiriyu, Oluwadamilola Samuel Alawode

**Affiliations:** Department of Economics, College of Social and Management Sciences, Tai Solarin University of Education, Ijagun, Ijebu Ode, 120101 Nigeria

**Keywords:** COVID-19 shock, Foreign direct investment, OECD countries, AMG estimator

## Abstract

The role of foreign direct investment flows in the growth and development of any nation cannot be overemphasized. However, different economic issues influence the pattern and flow of several investment channels. Notable among such economic crises is the recent COVID-19 pandemic that ravaged the entire global economy and restricted the flow of foreign investment among countries. With the perceived influence of the pandemic on businesses and investments, this study investigates the impact of COVID-19-related shock on the FDI flows of OECD countries. Using the Augmented Mean Group (AMG) long-run estimator, it reveals that the COVID-19 shock harms FDI inflows across OECD but enhances the outflows of FDI from OECD. Furthermore, the comparative analysis of the Eurozone and non-Eurozone countries in OECD shows that the effect of COVID-19 shock on FDI flows is positive in the former but otherwise in the latter. Hence, the monetary authorities of these countries must implement favorable monetary policies that will enhance new and ongoing investments as well as the expansion of industrial activities. Also, policymakers in this region should encourage the formulation of economic frameworks that are resilient to several global and country-specific economic uncertainties to safeguard the economies from unforeseen circumstances.

## Introduction

Following the Ebola virus epidemic which dominated the Western African countries from 2013 to 2016 and the Zika virus of 2015 through 2016, the world experienced another pandemic in the last quarter of 2019. The pandemic which was traced to Wuhan, a Chinese city, was identified to be a new variant of the virus called Coronavirus of 2019 (COVID-19). Since the virus is a transmissible disease, interaction among people increases its spread across the globe with high daily recorded new and death cases [1]. As of March 11^th^, 2020, the virus is declared a global pandemic by the World Health Organization [2]. At the initial stage of its outbreak, the virus was identified as a health crisis, but it was later classified as an economic crisis due to its adverse implication on several macroeconomic indicators such as national income, aggregate consumption, trade, savings, and investment.

In a bid to avert the shock posed by the pandemic, several government interventions were introduced, especially total lockdown, business closure, and international travel [3]. Despite these measures, COVID-19 has since its outbreak spread across over 200 countries touching every continent of the world. The occurrence of the pandemic invariably brought about global economic uncertainty which disrupts the global market. For instance, the lockdown policy which is stringent in some advanced countries hurts the global supply chains, particularly crude oil.

While there is no doubt that the COVID-19 shock brought about a decline in the world trade volume, investment, and other economic activities, the reduction in inter-country activities brought a drastic fall in the level of foreign investment given that it is one of the major drivers of the global economy through cross-country transfer of goods and services facilitation and provision of capital around the world. The adverse effect of the COVID-19 pandemic is felt by all the countries of the world especially the major top recipients of foreign direct investment (FDI) in the world such as China, the USA, and the UK [4, 5]. Meanwhile, it is observed that despite several government policies to revamp the global economy, the perceived pandemic-induced shock raised investors’ sentiment on FDI across the globe [6].

The outbreak and spread of COVID-19 attracted much scholarly research in an attempt to compare the behavior of several macroeconomic variables before and during the COVID-19 pandemic in order to predict the aftermath effect of the pandemic on the stability of different economies. For instance, Phan and Narayan [7] assess the pandemic impact on stock price, Gil-Alana and Monge [8] focus on its impact on oil price, whereas Narayan [9] based his study on the exchange rate, and Vidya and Prabheesh [10] investigate the impact of the virus on international trade. Meanwhile, recent studies [see [6, 11, 12] focus on the economic implication of the global pandemic on FDI flows. However, none of these studies considered the effect of the COVID-19 pandemic on FDI flows in OECD countries.

From the empirical survey, some studies [see [13–23] stress that FDI flows are often affected by uncertainties. This is similar to the findings reported by recent studies [24–28] that COVID-19 shock deteriorates FDI flows across the world except for China. However, the prediction of UNCTAD [4] suggests a drastic fall in the level of global FDI flows following the outbreak of the COVID-19; meanwhile, the global FDI statistics in 2021 doubled the value before the outbreak of the pandemic. Categorically, in the OECD area, FDI inflows in 2020 were 75% higher than the 2019 figure, while the 2021 statistic is twice the recorded value in 2020 with the USA and the UK being the top recipients of FDI [5, 28].

Following the empirical channel, it is clear that no study has assessed the effect of COVID-19 shock on FDI flows across OECD countries. To bridge this vacuum, this study examines the impact of COVID-19-related shock on the inflows and outflows of FDI in the OECD countries. These countries have been categorized in the literature as the industrialized and leading recipients of FDI inflows. Also, the majority of these countries are leading economies in terms of ground-breaking economic policies that facilitate access to the international market through a rule-based international framework for investment. Thus, the rationale for the investigation of the possible effect of COVID-19 shocks on their FDI flows.

Furthermore, this study partitions the group into Eurozone and non-Eurozone environments with the focus of providing an answer to the following questions: (i) do FDI inflows and outflows in the Eurozone and non-Eurozone countries react differently to COVID-19 shock? (ii) Are FDI inflows across the countries sensitive to COVID-19 shock than FDI outflows? This decision is motivated by two factors. First, the majority of the non-Eurozone countries are the top recipient of FDI inflows across the globe and these countries such as Canada, Japan, the UK, and the USA, constitute two-thirds of the G7 countries that were most affected by the outbreak of COVID-19 pandemic. Therefore, there is a possibility that the shock posed by the COVID-19 will be stronger in non-Eurozone countries than in its counterpart. Second, the adoption of a common currency platform by the European countries through the use of the Euro also serves as a major condition that facilitates the flows of FDI within the Eurozone countries compared to the non-Eurozone countries.

The final contribution of this study is methodological. In an attempt to develop the COVID-19-induced shock, this study creates a cross-sectional time-invariant dummy quarterly COVID-19-related shock. Hence, to circumvent the issue of cross-sectional dependence, non-stationarity, and heterogeneity, the effect of COVID-19 shock on FDI flows across OECD countries is examined through the application of the Augmented Mean Group (AMG) long-run estimator which is robust to the aforementioned statistical properties of the series.

The remaining parts of this study are organized as follows; “Review of related literature” section provides an empirical review of FDI flows before and during the COVID-19 pandemic. “Methodology” section describes the data as well as the model of the study, while “Results and Discussions” section focuses on the interpretation and discussion of findings and “Conclusion and Recommendation” section concludes and gives policy recommendations.

## Review of related literature

The benefits of FDI to the growth of any economy include but are not limited to economic development and stimulation, ease of access to the international market, employment generation, accumulation of resources, technology, and exchange of knowledge between the host and home countries. However, these potential benefits are often disrupted in the face of internal and external shocks (economic, financial, and political), and in recent times health-related crisis such as the COVID-19 pandemic. The empirical review of this study is in two strands, first, it explores studies that focus on the impact of different economic uncertainties, pandemic, shocks, and financial crises on FDI flows, while the second strand focuses the reaction of FDI flows to the outbreak of the COVID-19 pandemic.

## FDI amidst previous economic crises, shocks, and uncertainties

The proliferation of studies on the link between FDI flows and uncertainty adopts the economic policy uncertainty (EPU) index developed by Baker et al. [17]. However, some studies consider the effect of shocks on FDI outflow and inflow using different economic measures [see [20]]. Meanwhile, studies [see [29]] analyze the impact of geopolitical tensions and financial crises on FDI. In an attempt to strengthen existing literature, Ahir et al. [30] adopt the World Uncertainty Index (WUI) against the domestic uncertainty used by Nguyen et al. [19]. These studies conclude differently that the relationship between domestic uncertainty and FDI is positive but negative using WUI. In addition, Canh et al. [21] investigate the influence of EPU on the net foreign direct investment of twenty-one countries. Using the linear panel model, the study adds to the existing literature in twofold, first, country-specific EPU hurts FDI outflows, while the combination of country-specific and global EPU is positively related to net outflows of FDI. Contrarily, Gulen and Ion [16] and Drobetz et al. [18] argue that uncertainty has an adverse consequence on the net inflow of FDI in the host country.

Nguyen et al. [19] examine the relationship between EPU, derivative use, and firm-level FDI and conclude that the EPU differential between the home and the host country has a significant effect on FDI. It is also identified that firms at all levels make use of derivatives and options during different EPU and firms prefer to invest in the host country with a minimal or reasonable level of EPU to their home country. Using the EPU index developed by Baker et al. [17], some studies [13, 16, 17] conclude that there is a strong negative relationship between aggregate investment and uncertainty. From another view, Vujanović et al. [23] examine the effect of the global financial crisis on the FDI of the transition economies (Croatia and Slovenia) between the periods of 2006 to 2014. It concludes that FDI in these countries is a function of learning and technological upgrading; therefore, the government should implement policies to enhance technology and foster learning as these are cogent, especially during a turbulent period when investment and innovation fall due to capital flight. Similarly, some researchers state that severe natural disasters lower FDI inflow in emerging economies [see [14, 15]. A study by Oh et al. [22] on natural diseases and multinational corporations suggests that the entry and exit of the multinational corporations are based on the economic, political, and social–environmental stability because natural disasters or epidemics hamper the growth of FDI inflows.

## FDI and the COVID-19 pandemic

Despite the adverse effect of the COVID-19 pandemic experienced by different countries across the globe, there are sparse studies on the connection between the COVID-19 pandemic and FDI flows in OECD. However, some studies for instance Ajide and Osinubi [24] utilize cross-sectional data of forty-three countries with the ordinary least square and quantile regression estimators to unveil the evidence of a positive relationship between COVID-19 cases confirmed, COVID-19 issue-related death, and FDI outflow of the countries. Conversely, Duan, et al. [26] reveal evidence of a negative effect of the COVID-19 pandemic on foreign trade and outward FDI, although the study argues that the adverse effect is transitory. This result conforms with the evidence reported by Aysan et al. [25] that COVID-19 negatively affects the global foreign direct investment flows, but the curtailment and recovery of China in response to the pandemic is faster than the Western economies.

Fang et al. [27] deduce that global COVID-19 has a serious implication for the global economy as well as foreign direct investment flows, although the reverse is the case in China due to positive growth in her foreign direct investment flows compared to other developed countries. This is not farfetched since the country implements different stringent policies to combat the spread of the virus. Giofre [28] examines the capital flight of foreign investors in the developed country during and after the COVID-19 pandemic outbreak. He states that during any economic distress, the advanced economies are less affected compared to the growing nations. He further recommends that a strong financial liquidity reserve is advisable for the survival of emerging countries during any economic or financial turbulence. This recommendation is similar to the following studies that some emerging economies become more volatile during the economic crisis which often leads to capital flights to advanced nations [see [31–34].

In summary, Ajide and Osinubi [24] argued that there is a high tendency that the COVID-19 pandemic will adversely affect FDI flows across countries of the world. This is similar to the conclusion of OECD [35] which states that FDI inflow will drop by 30% in the year 2020 as a result of the policies taken by the government to curtail the spread of the virus. In response to the assertion and following sparse empirical literature on COVID-19 shocks and FDI flows, particularly for OECD countries, this study examines the connection between COVID-19 shocks and FDI flows of these countries. It further decomposes the group into Eurozone and non-Eurozone countries to differentiate the effect across sub-samples. Finally, it applies the AMG long-run estimator to evaluate the reaction of FDI flows to COVID-19 shocks due to the presence of non-stationarity, heterogeneity, and cross-sectional dependence in the panel series.

## Methodology

### Data

In this study, we examine the impact of COVID-19 shock on foreign direct investment flows across twenty-three (23) Organisation for Economic Co-operation and Development (OECD) countries. The group is further partitioned into Eurozone and non-Eurozone. For this purpose, we collect quarterly data on foreign direct investment inflows % of GDP (FDII) and outflows % of GDP (FDIO), real gross domestic product (RGDP) growth rate to measure market size, inflation rate (INFR) employed as economic stability measure, interest rate (INTR) used to measure growth prospects, real effective exchange rate (REER), and gross fixed capital formation (GFCF) growth rate as a proxy for investment. Dataset related to FDI, interest rate, and gross fixed capital formation was retrieved from the OECD website (via data.oecd.org); meanwhile, the Federal Reserve database (via fred.stlouisfed.org) is the source for the remaining series. While data on FDII, FDIO, RGDP, GFCF, and interest rate were collected in quarterly observations, INFR and REER were sourced in monthly frequency and the quarterly series were interpolated accordingly. Hence, the scope of the study covers the period from 2013:Q1 to 2021:Q2. Due to the novelty of the COVID-19 pandemic and the absence of an appropriate indicator to measure its shock in a quarterly observation, we develop a dummy variable^1^ that takes the value of 1 from the first quarter of 2020 following the WHO’s declaration and 0 before the period.

### Model

To achieve the desired objective of this study which is to examine the impact of COVID-19 shock on FDI flows in OECD countries, we consider the empirical framework of the previous studies on the determinants of FDI. Some studies [see [36–39]] suggest factors such as economic growth, interest rates, exchange rates, inflation rates, and gross fixed capital formation as determinants of foreign direct investment flows. Meanwhile, the intensity of the shock posed by the global pandemic has shown that the COVID-19 shock can be modeled as a determinant of FDI flows.^2^ Thus, we include the COVID-19 pandemic as a determinant of FDI flows across OECD countries. The baseline model for our empirical investigation is therefore stated as;

1 $$\begin{aligned} {\text{FDI}}_{it} = & \alpha_{0} + \alpha_{1} {\text{COVID}}_{it} + \alpha_{2} {\text{RGDP}}_{it} + \alpha_{3} {\text{INTR}}_{it} + \alpha_{4} {\text{REER}}_{it} + \alpha_{5} {\text{INFR}}_{it} + \alpha_{6} {\text{GFCF}}_{it} + \mu_{it} ; \\ & i = 1,2, \ldots ,N;t = 1,2, \ldots ,T \\ \end{aligned}$$

where FDI stands for the individual measure of FDI inflows (FDII) and outflows (FDIO), while COVID is the developed COVID-19 shock and RGDP, INTR, REER, INFR, and GFCF, respectively, represent the real gross domestic product, interest rates, real effective exchange rates, inflation rates, and gross fixed capital formation. $\mu$ stands for the error term.

Due to the peculiarity of the dataset at hand with statistical features of cross-sectional dependence, non-stationarity, co-integration, and inherent heterogeneity in the slope coefficients, the study applies the Augmented Mean Group (AMG) within the panel data framework to estimate the long-run impact of COVID-19 shock on FDI flows across OECD countries. The AMG long-run panel data estimator developed by Eberhardt and Bond [40] is appropriate when the panel series exhibit the aforementioned statistical attributes. Interestingly, the estimator disentangles the conditional impact of the independent variables from the unobserved common elements in the structured framework. Through this approach, Eberhardt and Bond [40] infix a dummy element to show the inscription of the unobserved factors in the level forms of the variables. Thus, they adopted two steps to carry out this process as follows;

Step 1:

2 $$\Delta y_{it} = \gamma_{i} + \vartheta_{i} \Delta x_{it} + \rho_{i} f_{t} + \mathop \sum \limits_{t = 2}^{T} \delta_{i} \Delta D_{t} + \varepsilon_{it} .$$

Step 2:

3 $$\hat{\vartheta }_{AMG} = N^{ - 1} \mathop \sum \limits_{i = 1}^{N} \hat{\vartheta }_{i}$$

where the determining variable in this specification is FDI flows, denoted as ${y}_{it}$; the regressors (COVID, RGDP, INTR, INFR, REER, and GFCF) are encapsulated in ${x}_{it}$. ${\vartheta }_{i}$ stands for the cross-section specific parameters, and ${f}_{t}$ is the unobserved heterogeneous common factor. The developed dummy variable ${D}_{t}$ is expressed with ${\delta }_{i}$ to explain the common dynamic process ${D}_{t}{\delta }_{i}$, while the standard AMG estimator is represented by ${\widehat{\vartheta }}_{AMG}$.

In the literature, it has been widely conceived that there is another variant to this technique known as the Common Correlated Effects Mean Group (CCEMG) developed by Pesaran [42] that can estimate series when there is a presence of correlation between the observed explanatory variables and the common factors [see [43, 44]]. Despite this uniqueness, the superiority of the AMG framework over the CCEMG estimator can be seen in this regard, first, the former provides meaningful economic interpretation to the unobserved factors within the panel framework as compared to the latter which only produces nuisance parameters. Second, the AMG estimator tends to be more flexible through its ability to yield a unit coefficient adjusted on the common dynamic framework. Thus, it is on this ground that this study considers the AMG estimator as the appropriate technique to examine the long-run impact of COVID-19 shock on FDI flows across OECD countries.

## Results and discussion

To understand the dynamic relationship of the variables utilized in this study, we begin the empirical analysis from the preliminary analysis of the series. This is important to elucidate the statistical properties of the series under consideration. In addition, it tells us the behavior of the series and also provides information about the appropriate method that suits the variables in question. In this regard, we first examine the descriptive analysis of the series using mean, maximum, minimum, standard deviation, and probability for the full sample and sub-samples. This is followed by the trend analysis of FDI inflows and outflows for Eurozone and non-Eurozone countries through graphical visualization of the series.

Table 1 summarizes the descriptive statistics of the variables for the full sample and sub-samples of the OECD countries under consideration. The result shows that the average value of FDII across non-Eurozone countries is greater than the mean value of inflows in the entire OECD and Eurozone countries. This is also similar to the case of FDIO where non-Eurozone countries have the highest outflows followed by the entire OECD estimate and Eurozone countries. Conversely, the average value of gross fixed capital formation growth rate in the Eurozone countries is greater than the case of non-Eurozone countries and the entire OECD. This is also similar to the interest rate of Eurozone countries which appear to have a negative mean value as against the positive average value of interest rates in the other samples. Meanwhile, there is evidence of an average decrease in the level of inflation rate in the full sample and Eurozone countries but otherwise in non-Eurozone countries. The average value of the real effective exchange rate in Eurozone countries seems to be higher compared to the entire OECD and non-Eurozone countries. Finally, it is also observed that the real gross domestic product that measures the value of market size across the samples shows that the overall economic output of non-Eurozone countries is huge relative to the entire OECD and non-Eurozone countries. This implies that the non-Eurozone countries, on average, experienced an increase in real economic output over time compared to the other samples.

**Table 1:** Descriptive statistics

Variables	Mean	Max	Min	Std. dev	J-B
*Full sample*					
FDII	6806.6360	24,016.0000	− 72,472.8400	21,686.6700	**26,100.5700**
FDIO	8283.1500	147,026.0000	− 131,117.9000	22,661.2400	**3875.4240**
GFCF	1.0222	161.1494	− 47.0552	8.3936	**972,579.8000**
INFR	− 0.8981	5.6615	− 586.5857	21.2410	**1,786,594.0000**
INTR	0.5386	8.5700	− 0.7767	1.6715	**2947.1550**
REER	97.5315	150.6433	69.0467	10.3944	**784.0347**
RGDP	1,091,676.0000	9,080,401.0000	4121.5000	1,928,503.0000	**1842.2570**
*Eurozone*					
FDII	2752.4950	77,203.4200	− 62,952.0900	12,031.9400	**2142.1480**
FDIO	5355.1000	86,066.2800	− 121,342.2000	16,260.6100	**3786.9910**
GFCF	1.0774	161.1494	− 47.0552	10.2238	**351,282.3000**
INFR	− 1.5924	5.6615	− 586.5857	28.2474	**3,206,433.0000**
INTR	− 0.1818	0.4867	− 0.5425	0.2609	**44.4344**
REER	98.4620	108.8833	86.8367	3.8861	**21.5617**
RGDP	157,186.5000	750,021.2000	4121.5000	215,389.0000	**227.2621**
*Non-Eurozone*					
FDII	12,077.0200	240,161.0000	− 72,472.8400	29,084.4500	**3998.8840**
FDIO	12,089.6100	147,026.0000	− 131,117.9000	28,520.4800	**650.8165**
GFCF	0.9503	26.2370	− 28.3373	5.1308	**1397.2410**
INFR	0.0045	0.0288	− 0.0091	0.0059	**45.3345**
INTR	1.4750	8.5700	− 0.7767	2.1891	**173.6939**
REER	96.3219	150.6433	69.0467	15.0556	**70.6120**
RGDP	2,306,512.0000	9,080,401.0000	372,766.5000	2,426,749.0000	**106.9761**

The bolded values in the last column of the table represent the significance level at 1%

In addition to the mean value of the variables, the maximum and minimum values show that the countries (full and sub-samples) experienced different values of FDII, FDIO, GFCF, INFR, INTR, REER, and RGDP over time. This is further confirmed by the value of standard deviation which shows that there is a huge deviation of the variables from their mean value except in the case of inflation for non-Eurozone countries and interest rates for Eurozone countries. Then again, the normality check is conducted through the use of Jarque-Bera statistics and its probability value shows that all the variables are not normally distributed either for the full sample or the sub-samples at a 1% level of significance.

**Fig. 1:**
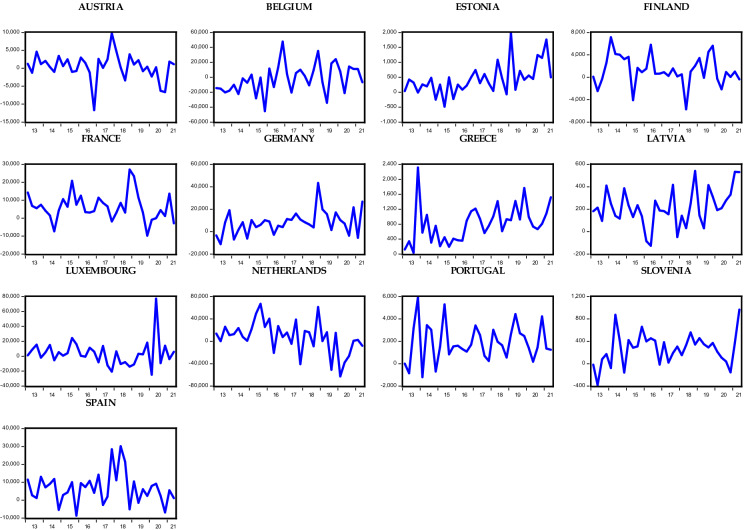
Trends in foreign direct investment inflows of Eurozone countries

**Fig. 2:**
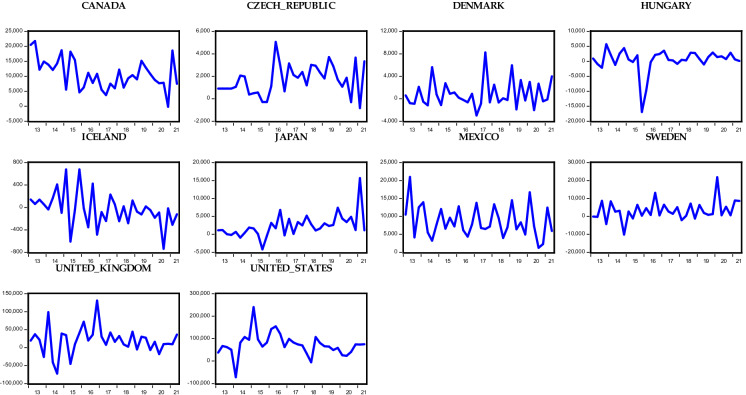
Trends in foreign direct investment inflows of non-Eurozone countries

**Fig. 3:**
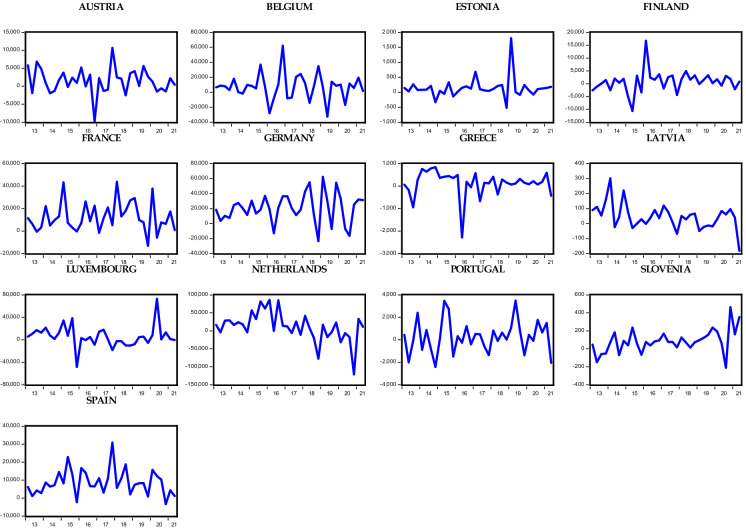
Trends in foreign direct investment outflows of Eurozone countries

**Fig. 4:**
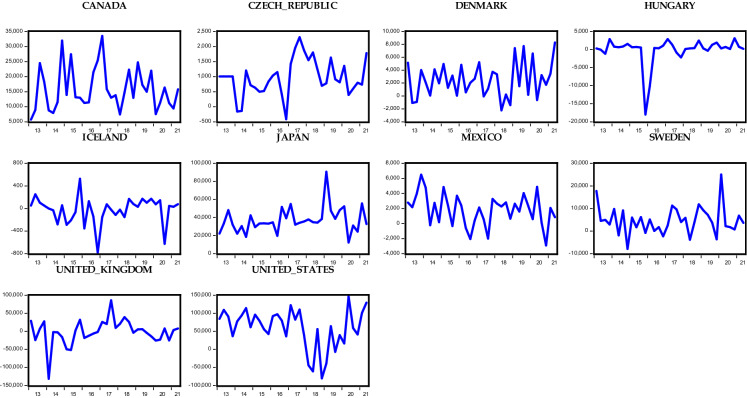
Trends in foreign direct investment outflows of non-Eurozone countries

Considering the OECD countries under consideration, Figs. 1, 2, 3, and 4 show the respective foreign direct inflows and outflows for the Eurozone and non-Eurozone countries. In the figures, the majority of the Eurozone and non-Eurozone countries experienced a high variability and fluctuation within the period examined. Meanwhile, Figs. 1 and 2 show that between the year 2016 and 2017, the majority of the countries experience a subdue inflow of foreign direct investment, which could be the aftermath effect of the oil price plunge in 2016 and different geopolitical events across the countries. These periods are followed by the recent COVID-19 pandemic. Starting from 2020, there is a huge drastic fall in the level of FDI inflow across both Eurozone and non-Eurozone countries due to lockdown and closure of borders as well as other stringent measures put in place to avert the spread of the virus. Surprisingly, FDI inflows trend upward in late 2021 for some Eurozone (Greece, Luxembourg, and Slovenia) and non-Eurozone countries (Czech Republic, Denmark, Iceland, and UK). This could be due to several policy responses adopted by the countries to revive their economy. However, in the case of FDI outflows in Figs. 3 and 4, only Slovenia experienced an improvement in her outflows among all other Eurozone countries, whereas the Czech Republic, Denmark, Iceland, UK, and the USA of America were the only countries across the non-Eurozone countries that improved their transfer of foreign investment to other countries. Therefore, it is safe to agree that despite the adverse effect of the COVID-19 pandemic on FDI inflows and outflows of OECD countries, some countries still experienced an improvement in the inflows and outflows of FDI.

Following the descriptive analysis, we test for the presence of cross-sectional dependence in the cross-sectional variables. The results based on the Breusch-Pagan LM, Pesaran scaled LM, adjusted scaled LM, and the Pesaran CD tests in Table 2 show that the variables exhibit cross-sectional dependence across the full sample and sub-samples. Hence, this informs the choice of using second-generation unit root tests, namely cross-sectional augmented Im, Pesaran, and Shin (CIPS), and cross-sectional augmented Dickey–Fuller (CADF), which as robust to cross-sectional dependence in panel series.

**Table 2:** Cross-sectional dependence test results

Variables	BP-LM	PS-LM	Adj-LM	P-CD
*Full sample*				
FDII	338.7***	3.8113***	3.4628***	1.1428
FDIO	288.8*	1.5945	1.2460	2.9705**
RGDP	6074.8***	258.81***	258.46***	76.226***
INF	2080.58***	81.243***	80.895***	35.709***
INR	4950.30***	208.82***	208.47***	45.975***
REER	2717.5***	109.56***	109.21***	24.133***
GCFC	1531.71***	56.845***	56.497***	28.6110***
*Eurozone*				
FDII	127.00***	3.9233***	3.7267***	1.4301
FDIO	77.245	− 0.0603	− 0.2573	2.5566**
RGDP	1851.03***	141.95***	141.79***	41.810***
INF	803.40***	58.078***	57.881***	57.881***
INR	2638.69***	205.019***	204.823***	51.3673***
REER	1179.55***	88.195***	87.1925***	29.2984***
GCFC	493.275***	33.2488***	33.051***	16.3106***
*Non-Eurozone*				
FDII	34.7650	− 1.0788	− 1.2303	− 0.8739
FDIO	47.653	0.2797	0.1281	1.1097
RGDP	1065.20***	109.647***	109.495***	32.4822***
INF	287.46***	25.4557***	25.406***	11.8891***
INR	480.667***	45.923***	45.7719***	7.1724***
REER	434.798***	41.088***	40.936***	− 0.01239***
GCFC	249.69***	21.5766***	21.4251***	11.8198***

BP-LM, PS-LM, Adj-LM, and P-CD, respectively, denote Breusch-Pagan LM, Pesaran scaled LM, adjusted scaled LM, and the Pesaran CD. ***, **, and * denote significance at 1%, 5%, and 10% level

The two second-generation unit root tests (CIPS and CADF) in Table 3 show that majority of the variables are significant at the level using CIPS, whereas they are non-stationary for the CADF test. This implies that the variables under consideration have a mixed order of integration; thus, there is a need to verify whether the series are cointegrated. Hence, this study adopts the combination of both first- and second-generation co-integration tests designed, respectively, by Kao [45] and Westerlund [46]. This becomes necessary to verify whether the variables are co-integrated in the presence of cross-sectional dependence.

**Table 3:** Unit root test

Variables	Full sample	Sub-sample		Full sample	Sub-sample	
		Eurozone	Non-Eurozone		Eurozone	Non-Eurozone
	CIPS			CADF		
FDII	− 5.098***^a^	− 4.947***^a^	− 5.398***^a^	− 2.636***^b^	− 2.931***^a^	− 2.276***^a^
FDIO	− 4.887***^a^	− 4.951***^a^	− 4.701***^a^	− 2.458***^b^	− 2.894***^a^	− 3.102***^a^
RGDP	− 4.956***^b^	− 4.713***^b^	− 4.668***^b^	− 2.716***^b^	− 2.759***^b^	− 2.779***^b^
INF	− 4.683***^b^	− 4.854***^a^	− 5.239***^a^	− 2.031***^b^	− 3.823***^a^	− 2.693***^a^
INR	− 2.711***^a^	− 6.190***^a^	− 2.706***^a^	− 2.068***^b^	− 6.190***^a^	− 2.526***^b^
REER	− 5.084***^b^	− 5.532***^b^	− 4.712***^b^	− 2.187***^b^	− 4.640***^b^	− 2.790***^b^
GCFC	− 5.977***^b^	− 5.586***^a^	− 5.354***^a^	− 2.654***^b^	− 4.919***^b^	− 3.831***^a^

^***^, **, and * represent the significance of the unit root model at 1, 5, and 10% level. a and b denote the level and first difference stationarities of the series, respectively

The results of the Kao residual-based cointegration test and the Westerlund cointegration test are presented in Table 4. The result of the Westerlund [46] is presented in Panel A, while Panel B documents the Kao [45] residual-based cointegration test. The result of the Westerlund cointegration test shows that the variables either in the full or sub-samples are cointegrated at a 1% level of significance. Meanwhile, the Kao residual-based cointegration test reveals that the models for both the full sample and Eurozone countries are cointegrated at 5%, while non-Eurozone countries are highly significant at a 1% level.

**Table 4:** Panel cointegration tests

Statistics	Full sample	Eurozone	Non-Eurozone	Full sample	Eurozone	Non-Eurozone
	FDII			FDIO		
*Panel A: Westerlund *[46]						
G_t_	− 3.081***	− 3.575***	− 4.095***	− 4.305***	− 4.311***	− 4.298***
G_a_	− 30.310***	− 29.435***	− 31.318***	− 33.322***	− 33.528***	− 33.072***
P_t_	− 19.070***	− 14.754***	− 11.802***	− 18.175***	− 17.007***	− 11.414***
P_a_	− 33.770***	− 35.131***	− 30.959***	− 30.096***	− 33.694***	− 24.423***

	*t*-statistics			*t*-statistics		
*Panel B: Kao *[45]						
ADF	− 1.7864**	− 2.0875**	− 4.1148***	− 2.0702**	− 1.7136**	− 4.0320***
Residual Variance	86.3960	92.9420	− 72.8580	81.6870	94.1830	63.7808
HAC Variance	10.6840	13.1410	17.3038	11.6675	11.9070	18.7043

^***^, **, and * represent the significance of the unit root model at the 1, 5, and 10% levels

Following the preliminary analyses of the study, it is evident that despite the presence of cross-sectional dependence and non-stationarity in the panel series, the variables are cointegrated. Thus, the appropriate technique following evidence of the highlighted statistical attributes is the Augmented Mean Group (AMG) estimator designed by Eberhardt and Bond [40]. The long-run estimator is adopted having confirmed the preliminary condition that warrants its usage for panel analysis.

For the main analysis, the study reports the estimates of the variables for foreign direct investment inflows and outflows in separate tables for full sample and sub-samples. In the case of FDI inflows, the results of the AMG estimator are presented in Table 5, while Table 6 covers the results for FDI outflows for the sampled categories. Table 5 shows the effect of COVID-19, real gross domestic product, inflation, interest rate, real effective exchange rate, and gross fixed capital formation on the FDII of the OECD countries between the years 2013:Q1 and 2021:Q2. The result shows that there is a negative relationship between the COVID-19 pandemic and the FDI inflow of the OECD countries. This implies that an increase in the COVID-19 pandemic will lead to a fall in FDII by 0.0117 though not significant. The other macroeconomic variables show that there is a positive relationship between RGDP, INT, REER, and GFCF on FDII among the OECD countries. This means that the higher the RGDP, INT, REER, and GFCF, the greater the inflow of FDI in OECD. Thus, this result conforms with the argument of OECD [35]. Furthermore, Srinivasan et al. [47] and Quoc and Thi [48] state that there is a positive causality between GDP and FDI.

**Table 5:** Long-run estimation results of OECD countries

Variables	Full sample		Sub-sample			
			Eurozone		Non-Eurozone	
Foreign Direct Investment Inflows (FDII)						
	Coefficient	Std Error	Coefficient	Std Error	Coefficient	Std Error
COVID-19	− 0.0117	0.0521	0.0169	0.0922	− 0.0028	0.0049
RGDP	0.1349	0.0756*	0.2102	0.1319	− 0.0319	0.0232
INF	− 1.170	2.018	− 1.231	3.451	− 0.1507	0.1855
INR	0.0613	0.0781	1.097***	0.1375	− 0.0072	0.0090
REER	0.2605	0.9635	− 2.166	1.710	− 0.1345	0.1069
GFCF	0.0039	0.0016*	0.0064**	0.0029	0.0003	0.0004
Constant	− 0.0253	0.0279	− 0.2685***	0.0476	0.0177*	0.0101
Diagnostic						
Wald Test	46.58***		1150.40***		16.88***	

^***^, **, and * represent the significance of the unit root model at the 1, 5, and 10% levels

**Table 6:** Long-run estimation results of OECD countries

	Full sample		Sub-sample			
			Eurozone		Non-Eurozone	
Foreign Direct Investment outflows (FDIO)						
	Coefficient	Std Error	Coefficient	Std Error	Coefficient	Std Error
COVID-19	0.0046	0.0855	0.0167	0.1519	− 0.0144***	0.0039
RGDP	0.1366	0.1677	0.1462	0.3136	0.0276	0.0262
INF	− 0.3381	1.697	0.0153	2.8950	0.5924	0.6346
INR	0.0661	0.1232	0.8622***	0.2160	− 0.0147	0.0097
REER	− 0.7751	0.8060	− 2.9380**	1.3870	− 0.1041*	0.4513
GFCF	0.0043	0.0011	0.0084**	0.0025	− 0.0036	0.0003
Constant	0.0292	0.0376	− 1.4380**	0.0639	0.0267**	0.0081
*Diagnostic*						
Wald Test	123.70***		5690.97***		75.41***	

^***^, **, and * represent the significance of the unit root model at the 1, 5, and 10% levels

Consider the two sub-samples which are Eurozone and non-Eurozone countries. In the Eurozone region, an increase in the COVID-19-related shock has a positive effect on the inflows of FDI. This implies that despite the COVID-19 pandemic that ravaged the global economy, the Eurozone countries continue to attract inflows of foreign investment into the region. This is supported by Ajide and Osinubi [24]. However, the real gross domestic product, interest rate, and gross fixed capital formation are positively related to FDI inflow. This result is not farfetched because investors are sensitive to the growth rate and the prevailing interest rate of any economy since these factors determine the growth prospect of the nations. Also, an accelerated gross fixed capital formation of a country determines the decision of the investors either to invest or not. Meanwhile, inflation which determines economic stability along with real effective exchange affects the earnings of the investor based on evidence of negative influence on the inflow of FDI in the Eurozone region.

For the non-Eurozone countries, there is a negative relationship between COVID-19 and FDII. Likewise, RDGP, INF, INR, and REER hurt FDI inflows. Avom et al. [49] state the effect of COVID-19 on FDI inflow is higher in emerging economies like non-Eurozone countries as compared to the advanced countries. In addition, Hsieh et al. [20] confirm the existence of a negative relationship between COVID-19 and FDI inflow. In the work of Ho and Gan [50], the negative effect of the global pandemic is higher and greater in the emerging markets and this affects the growth prospect in this region. The result of the GCFC shows that there is a positive relationship between FDII and COVID-19. This conforms with the argument of Soylu [51] that for the growth of FDII to be sustainable, there must be strong savings and capital formation.

As regards the estimation of FDIO for the full and sub-samples, the result is presented in Table 6. The result shows that across the OECD countries under consideration there is a positive relationship between COVID-19 and the outflow of investment in OECD countries. This affirms the conclusion of OECD [35] that FDI outflows are expected to increase by 11% in the third quarter of 2021 compared to the previous years. Notable countries in the OECD that experienced more outflow of FDI are Japan, the Netherlands, and USA. The other variables except INF and REER exhibit a positive relationship with FDIO, while INF and REER have a negative relationship with FDIO. This implies that factors like RGDP, GFCF, and INTR were the factors that contributed to the outflows of FDI from OECD countries despite the adverse shock posed by COVID-19.

In the case of the Eurozone, COVID-19 contributed positively to the outflow of FDI implying that an increase in the spread of COVID-19 leads to an increase of 0.016 in FDIO. Also, RGDP, INF, INR, and GFCF are part of the major factors affecting the outflow of FDI among these countries as they are positively related to FDIO except REER. Conversely, the COVID-19 shock negatively affects the outflows of FDI in non-Eurozone countries which affirms the conclusion of OECD [5] that the non-Eurozone FDI flows will be more affected by COVID-19. As regards the other variables, only RGDP and INF have a positive contribution to the outflows of FDI across the non-Eurozone countries. The findings corroborate the conclusion of Al-thaqeb and Algharabali [29]; Ahir et al. [30] and Drobetz et al. [18] that both the economic, political, and pandemic uncertainties are negatively related to the foreign direct investment flows of the host countries.

## Conclusion and recommendation

The outbreak of the global COVID-19 pandemic brought about a severe economic condition that affected global economic activities, leading to the fluctuation in several economic indicators. The shock associated with COVID-19 significantly affects global investment and deteriorates the economic activities of countries in different capacities. Following the review of empirical studies on the determinants of FDI flows, it is observed that past and recent studies are yet to examine the effect of COVID-19-induced shock on FDI flows in OECD.

Based on this background, this study applies the AMG long-run estimator given the presence of cross-sectional dependence, non-stationarity, and cointegration among the variables. In this regard, it considers the impact of dummy COVID-19 shock, real gross domestic product, gross fixed capital formation, interest rate, inflation rates, and real effective exchange rate on FDI inflows and outflows of the OECD countries. In addition, this study partitioned the sample OECD countries into Eurozone and non-Eurozone countries following the conclusion that the extent of currency disparity between the groups will contribute significantly to the flows of FDI, particularly during the COVID-19 pandemic.

Results from the full sample show that FDI inflow in the OECD is adversely affected by COVID-19 shock, while the shock has consequently enhanced FDI outflow in the region. Our analyses further show that improvement in majority economic indicators such as real gross domestic product, interest rate, exchange rate, and gross capital formation has attracted foreign investment in the OECD based on the evidence of their positive impact on FDI flows. However, while the real gross domestic product, interest rate, and gross capital formation were found to have a positive relationship with FDI outflow, inflation and exchange rate were not determinants of outflows in the OECD. In the sub-region analysis, we discover that the reaction of FDII to COVID-19 shock is positive i.e., the inflows of FDI increase in the Eurozone region despite the COVID-19 effect on economies. However, it disrupts the inflow of FDI in the non-Eurozone region. While the former contrast with the projection of the UNCTAD [4], the latter conforms with the argument. Our analyses also show that the COVID-19 shock positively contributed to the outflow of FDI in the Eurozone and affected the non-Eurozone negatively.

Furthermore, the comparative analysis of the two sub-regions in the OECD shows that FDII in the Eurozone countries responds positively to real gross domestic product, interest rate, and gross fixed capital formation, whereas all economic indicators except gross capital formation were negatively related to FDII in the non-Eurozone. From the outflow analysis, we discover a positive relationship among other economic variables and FDIO except exchange rate in the Eurozone with most of these variables negatively related to FDIO in the non-Eurozone except gross domestic product and inflation.

Based on the above findings, the policymakers must act fast to revert and mitigate the effect of the global pandemic on the flows of FDI among the OCED countries especially the non-Eurozone where the COVID-19 shock adversely affected their FDII. The reduction in FDII can be averted through the development of favorable monetary policies to attract investors as well as the expansion of industrial activities. Also, an economically stable economy gives investors the courage to channel foreign investment; thus, policymakers in the Eurozone and non-Eurozone countries must pay attention to the level of inflation rate as this predicts the future of off-shore investments. In addition, policymakers in the OCED region should encourage the formulation of economic frameworks that is resilient to several global and country-specific economic uncertainties. In conclusion, further studies should focus on the country-specific analysis of the determinants of FDI flows in OECD during the global pandemic.

## Data Availability

All the data utilized in the study are publicly available at the referenced sources in methodology section.

## Appendix

### Appendix 1: List of selected OECD countries

**Table:**

S/N	Eurozone	Non-Eurozone
1	Austria	Canada
2	Belgium	Czech Republic
3	Estonia	Denmark
4	Finland	Hungary
5	France	Iceland
6	Germany	Japan
7	Greece	Mexico
8	Latvia	Sweden
9	Luxembourg	UK
10	Netherlands	USA
11	Portugal	
12	Slovenia	
13	Spain	

### Appendix 2: Data description and sources

**Table:**

Variables	Measurement	Source
Foreign Direct Investment Inflows (FDII) % of GDP	Foreign Direct Investment inflow divided by real gross domestic product	OECD Database (data.oecd.org)
Foreign Direct Investment outflows (FDIO) % of GDP	Foreign Direct Investment outflow divided by real gross domestic product	OECD Database (data.oecd.org)
Real Gross Domestic Product (RGDP) growth rate	Real Gross Domestic Product at current quarter minus Real Gross Domestic Product at previous quarter divided by Real Gross Domestic Product at the previous quarter	Federal Reserve database (fred.stlouisfed.org)
Inflation Rate (INFR)	Consumer Price Index of All Items at current quarter minus Consumer Price Index of All Items at previous quarter divided by Consumer Price Index of All Items at the previous quarter	Federal Reserve database (fred.stlouisfed.org)
Interest Rate (INTR)	Long-term interest rates are generated as percentages of averages of daily rates	OECD Database (data.oecd.org)
Real Effective Exchange Rate (REER)	Natural logarithm of Real effective exchange rate measured as nominal exchange rate (a measure of the value of a currency against a weighted foreign currency) divided by price deflator	Federal Reserve database (fred.stlouisfed.org)
Gross Fixed Capital Formation (GFCF) growth rate	Gross fixed capital formation in the current quarter minus Gross fixed capital formation in the previous quarter divided by Gross fixed capital formation in the previous quarter	OECD Database (data.oecd.org)
COVID-19 shock	It takes a value of 0 s from 2013Q1 to 2020Q1 and 1 s thereafter	Authors’ development

## Abbreviations

AMG: Augmented mean group
CADF: Cross-sectional augmented Dickey–Fuller
CCEMG: Common correlated effects mean group
CEE: Central and Eastern Europe
CIPS: Cross-sectional augmented Im, Pesaran, and Shin
COVID-19: Coronavirus of 2019
EPU: Economic policy uncertainty
FDI: Foreign direct investment
OECD: Organisation for Economic Co-operation and Development
UNCTAD: United Nations Conference on Trade and Development
WHO: World Health Organisation
WUI: World Uncertainty Index

## Footnotes

1 Similar indicator is developed by Barbero et al. [41] to examine the impact of COVID-19 shock on trade.

2 See Duan et al. [26], Fu et al. [6]; Sharma [11] for empirical survey on impact of COVID-19 on FDI.
